# Vialinin A Inhibits Microtubule-Mediated Secretory Granule Transport for Degranulation in RBL-2H3 Cells by Targeting the PI3K/Akt Axis

**DOI:** 10.3390/molecules31162825

**Published:** 2026-08-13

**Authors:** Ange Murielle Djidjou Tagne, Kotoe Ishii, Yasukiyo Yoshioka, Kouichi Sugaya, Jun-ichi Onose, Shunsuke Yajima, Naoki Abe

**Affiliations:** 1 Department of Nutritional Science and Food Safety, Faculty of Applied Bioscience, Tokyo University of Agriculture, 1-1-1 Sakuragaoka, Setagaya-ku, Tokyo 156-8502, Japan; 10822001@nodai.ac.jp (A.M.D.T.); 10725001@nodai.ac.jp (K.I.); k3sugaya@nodai.ac.jp (K.S.); j1onose@nodai.ac.jp (J.-i.O.); 2 Department of Health and Nutrition Sciences, Faculty of Human Health, Komazawa Women’s University, 238 Sakahama, Inagi-shi, Tokyo 206-8511, Japan; y-yoshioka@komajo.ac.jp; 3 Department of Bioscience, Faculty of Life Sciences, Tokyo University of Agriculture, 1-1-1 Sakuragaoka, Setagaya-ku, Tokyo 156-8502, Japan; yshun@nodai.ac.jp

**Keywords:** vialinin A, antiallergic activity, microtubule, Fyn, PI3K

## Abstract

The bioregulatory function of food-derived bioactive factors in complementary therapies for lifestyle-related diseases is attracting worldwide attention. Vialinin A, a *p*-terphenyl compound isolated from the edible Chinese mushroom *Thelephora vialis*, inhibits the production and release of tumor necrosis factor α and Syk kinase activity, a key protein involved in the development of allergic rhinitis. However, its effect on other calcium-independent signaling molecules remains unclear. This study investigated the effects of vialinin A on microtubule formation signaling pathways in RBL-2H3 cells. The results showed that vialinin A inhibited Fyn kinase activity and reduced PI3K (class IA) phosphorylation in a dose-dependent manner. Notably, vialinin A inhibited PI3Kδ kinase activity and reduces Akt phosphorylation even at low concentrations. These findings suggest that vialinin A suppresses microtubule formation and granule translocation required for mast cell degranulation through inhibition of the Fyn–PI3K–Akt signaling pathway. Collectively, vialinin A may represent a promising lead compound for the development of anti-allergic therapeutics.

## 1. Introduction

Aggregation of IgE-bound surface receptors (FcεRI) on mast cells following antigen stimulation initiates a series of receptor-proximal and distal signaling events responsible for type I hypersensitivity [1,2]. Some of the receptor-proximal events that occur in response to FcεRI aggregation include the transphosphorylation of immunoreceptor tyrosine-based activation motifs by Lyn, a Src family protein tyrosine kinase; the recruitment and activation of Syk kinase; and the activation of associated downstream signaling molecules such as the linker for activation of T cells (LAT) and phospholipase C-γ (PLCγ) [3,4,5,6]. These events result in increased intracellular Ca^2+^ concentration, leading to mast cell degranulation through the calcium-dependent pathway. In contrast, degranulation requires activation of a calcium-independent pathway that transports granules to the plasma membrane via microtubule formation via the Fyn-Gab2-PI3K signaling pathway [7,8,9,10]. This route involves the activation of Fyn, another Src family protein tyrosine kinase, which in turn induces the activation of Grb2-associated binding protein 2 (Gab2). Gab2 activates RhoA, a small GTPase member of the Ras family, which then initiates microtubule formation, and phosphatidylinositol 3-kinase (PI3K), which ensures signal amplification through the recruitment of Bruton’s tyrosine kinase, a TEC family kinase. These events result in cytoskeletal reorganization, which facilitates the translocation of secretory granules to the plasma membrane for degranulation. Proteases, vasoactive amines such as histamine, and lysosomal enzymes such as ꞵ-hexosaminidase [11,12,13] are often released within minutes of antigen sensitization. Within several hours, degranulation may be followed by the synthesis and secretion of cytokines such as tumor necrosis factor *α* (TNF-α) and chemokines that regulate inflammatory responses [14,15].

Recently, alternative medical therapies have received considerable attention because of their limited side effects compared to conventional therapies [16,17,18]. Functional foods have been of great interest because they provide readily available support for boosting immunity during global health crises, such as the recent coronavirus disease 2019 pandemic, when healthcare institutions were overcrowded [19,20]. We previously reported that vialinin A, a molecule isolated from the popular edible Chinese mushroom *Thelephora vialis*, is a potent inhibitor of the late-phase cytokine TNF-α [21,22,23]. In addition, we demonstrated that vialinin A potently inhibits antigen-induced mast cell degranulation, as evaluated by β-hexosaminidase release, with an IC_50_ of 0.25–0.4 µM. To elucidate its mechanism of action, we investigated the calcium-dependent signaling pathway and found that vialinin A inhibited the autophosphorylation and kinase activity of Syk, resulting in suppression of downstream LAT and PLCγ signaling [24]. However, the inhibitory effects on this pathway alone appeared insufficient to account for the potent inhibition of degranulation observed at low concentrations of vialinin A. This discrepancy led us to hypothesize that vialinin A may additionally regulate the calcium-independent pathway responsible for microtubule-dependent secretory granule transport, which has emerged as an essential process in mast cell degranulation. In the present study, we demonstrate that vialinin A suppresses activation of the Fyn–PI3K–Akt signaling axis, thereby inhibiting microtubule track formation required for secretory granule transport. These findings reveal a previously unrecognized mechanism of action of vialinin A and suggest that its potent anti-degranulation activity results from coordinated inhibition of both calcium-dependent and calcium-independent signaling pathways.

## 2. Results

### 2.1. Vialinin A Inhibited the Activation of Fyn and PI3K Kinases

**Figure 1:**
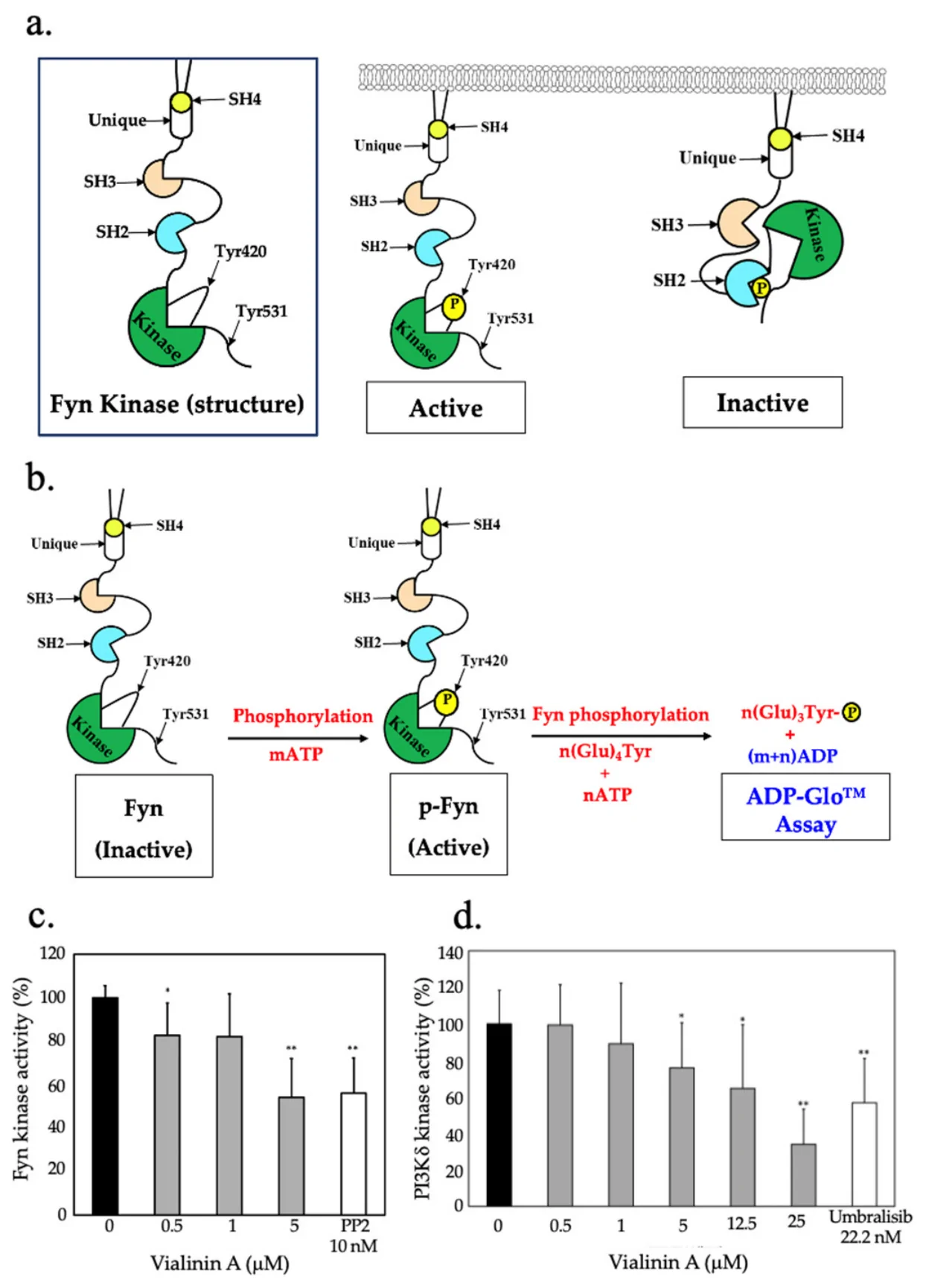
Fyn structure and effect of vialinin A on its kinase activity. (**a**) Fyn kinase domain organization. (**b**) Mechanism of Fyn kinase activity measurement using an ADP-Glo^TM^ kit. (**c**) Measurement of newly synthesized ATP following Fyn kinase reaction using (Glu)_4_Tyr as a substrate in the presence of vialinin A. PP2 (10 nM) was used as a positive control. (**d**) Measurement of newly synthesized ATP following PI3Kδ kinase reaction using PI(4,5)P_2_ as a substrate in the presence of vialinin A. Umbralisib (22.2 nM) was used as a positive control. Data are presented as mean ± SD of three independent experiments performed in triplicate. Asterisks indicate significant differences from the blank without inhibitor (0 µM), * *p* < 0.05 and ** *p* < 0.01.

Fyn tyrosine kinase (Figure 1a) is a key initiator in the calcium-independent microtubule-dependent signaling pathway of mast cell degranulation. The effect of various concentrations of vialinin A on Fyn kinase enzymatic activity against PP2, a positive control, was investigated using a Fyn kinase reaction kit, following the steps shown in Figure 1b. The results showed that, compared to the negative control (dimethyl sulfoxide, DMSO), vialinin A inhibited the activation of Fyn kinase in a dose-dependent manner, showing approximately 50% inhibition at 5 μM (Figure 1c).

Furthermore, as shown in Figure 1d, vialinin A inhibited PI3Kδ kinase activity in a concentration-dependent manner. The inhibitory activity was approximately 11%, 24%, 35%, and 66% at concentrations of 1.0, 5.0, 12.5, and 25 μM, respectively. Statistically significant inhibition was observed at concentrations of 5.0 μM and above, with inhibition reaching 66% at 25 μM.

### 2.2. Molecular Docking Analysis of Vialinin A with Fyn, Lyn, and PI3Kδ

**Figure 2:**
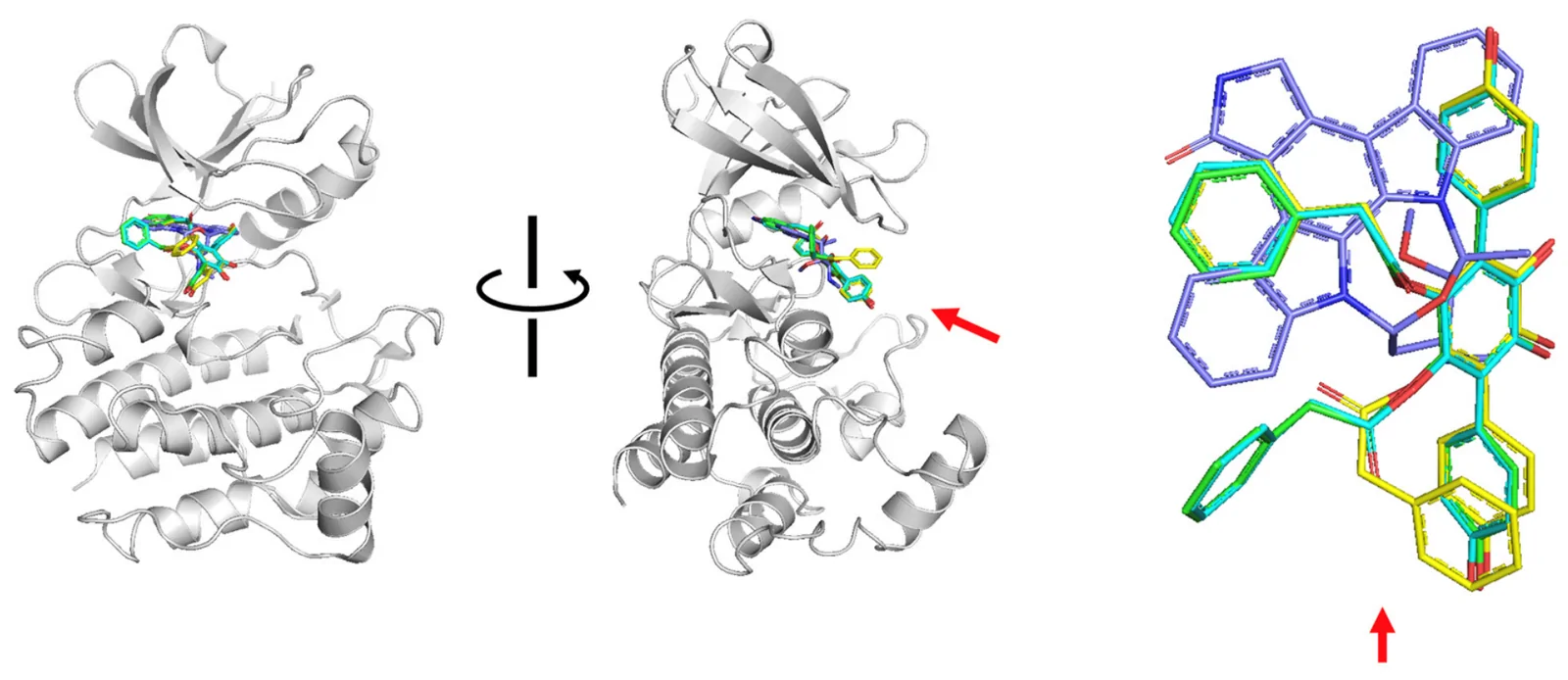
Superposition of the top three docking models of vialinin A with the crystal structure of the Fyn–staurosporine complex. The top three docking models of vialinin A are shown superimposed on the crystal structure of Fyn kinase complexed with staurosporine (PDB ID: 2DQ7). Front and side views are presented in the left and middle panels, respectively, with staurosporine and vialinin A displayed as stick representations. Staurosporine is colored purple, whereas vialinin A is colored cyan, green, and yellow in order of decreasing predicted binding affinity. The right panel shows only the bound molecules. Red arrows in the middle and right panels indicate the viewing direction of the compound.

Vialinin A was docked into the ATP-binding site of Fyn kinase using AutoDock Vina ver.1.2.3 [25]. The predicted binding affinities of the top three docking models were −9.3, −9.1, and −9.1 kcal/mol. As a control, staurosporine was also subjected to docking analysis, yielding the highest predicted binding affinity of −12.4 kcal/mol. All three docking models placed vialinin A within the ATP-binding site of Fyn kinase. Superposition of the docked vialinin A models with the crystal structure of the Fyn–staurosporine complex (PDB ID: 2DQ7) showed that vialinin A occupied a binding position similar to that of staurosporine (Figure 2). Although vialinin A and staurosporine occupied similar positions within the ATP-binding pocket of Fyn kinase, the three docking models of vialinin A exhibited distinct binding modes and interacted with different sets of amino acid residues (Supplementary Figure S1).

In the case of Lyn kinase, the predicted binding affinities of the top three docking models were −9.3, −9.3, and −9.0 kcal/mol, whereas staurosporine exhibited a highest predicted binding affinity of −11.4 kcal/mol. The docked vialinin A molecules occupied the ATP-binding site and largely overlapped with the binding position of staurosporine (Supplementary Figure S2). Although vialinin A and staurosporine occupied similar positions within the ATP-binding pocket, they were predicted to interact with different sets of amino acid residues, as observed for Fyn kinase. In addition, the detailed binding mode of vialinin A differed between Fyn and Lyn kinases, despite their similar predicted binding affinities (Supplementary Figure S3).

Docking analysis of vialinin A was also performed using PI3Kδ. The predicted binding affinities of the top three docking models were −7.7, −7.7, and −7.6 kcal/mol. In contrast, docking analysis of idelalisib yielded predicted binding affinities of −9.3, −8.2, and −8.1 kcal/mol for the top three models. The predicted binding site of vialinin A was located in a cavity distinct from the idelalisib-binding site observed in the crystal structure (Supplementary Figure S4; Site 1). When idelalisib was subjected to docking analysis, the top-ranked model occupied essentially the same position as that observed in the crystal structure (Supplementary Figure S4; Site 2). In contrast, the second-ranked model was located in a position similar to that predicted for vialinin A (Site 1), whereas the third-ranked model occupied a distinct site (Supplementary Figure S4; Site 3) that differed from both the vialinin A-binding site and the crystal structure-derived idelalisib-binding site.

### 2.3. Vialinin A Inhibited the Phosphorylation of PI3K/AKT Signaling Molecules in DNP-IgE/DNP-BSA Stimulated RBL-2H3 Cells

**Figure 3:**
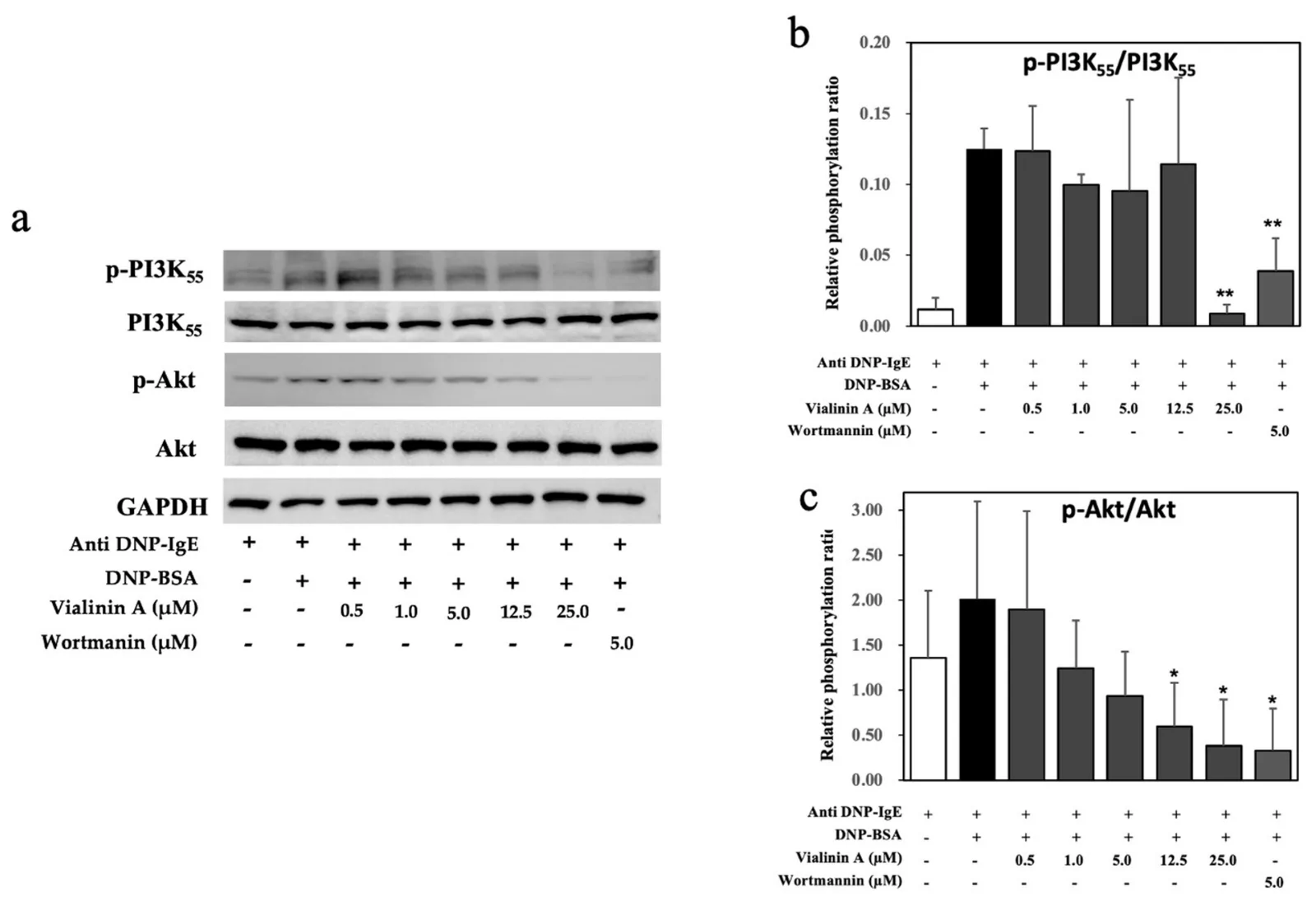
Effect of vialinin A on PI3K/AKT signaling pathways in DNP-IgE/DNP-BSA-stimulated RBL-2H3 cells. RBL-2H3 cells were sensitized with DNP-IgE for 16 h and treated with various concentrations of vialinin A for 10 min. Cells were then stimulated with DNP-BSA for 5 min, and the proteins were collected and quantified using Western blotting. (**a**) Phosphorylation of PI3K and AKT, shown as a representative Western blot from three independent experiments. (**b**) Densitometric analysis of p-PI3K_55_/PI3K_55_, and (**c**) Densitometric analysis of p-AKT/AKT. Data is presented as mean ± SD of three independent experiments. Dunnett’s multiple comparison test was used to evaluate statistically significant differences against the control group (wortmannin, 5 µM). Statistical significance was set at * *p* < 0.05 and ** *p* < 0.01.

PI3K and AKT are downstream signaling molecules of Fyn that are involved in the activation of the calcium-independent, microtubule-dependent signaling pathway. Thus, their phosphorylation is crucial for mast cell activation, resulting in degranulation. We therefore investigated the effect of vialinin A on the phosphorylation of these proteins using Western blotting. As shown in Figure 3, vialinin A inhibited the phosphorylation of PI3K and Akt following antigen stimulation in a dose-dependent manner. PI3K phosphorylation at Tyr199 on p55 was inhibited at concentrations of 25.0 μM, and this inhibition was expected to be dependent on the inhibition of Fyn kinase activity (Figure 3b). In contrast, as shown in Figure 3b, an inhibitory effect on Akt phosphorylation at Ser473 was observed, even at a low concentration (1.0 μM).

### 2.4. Vialinin A Inhibited Microtubule Formation and Its Translocation to the Plasma Membrane in DNP-IgE/DNP-BSA Stimulated RBL-2H3 Cells

**Figure 4:**
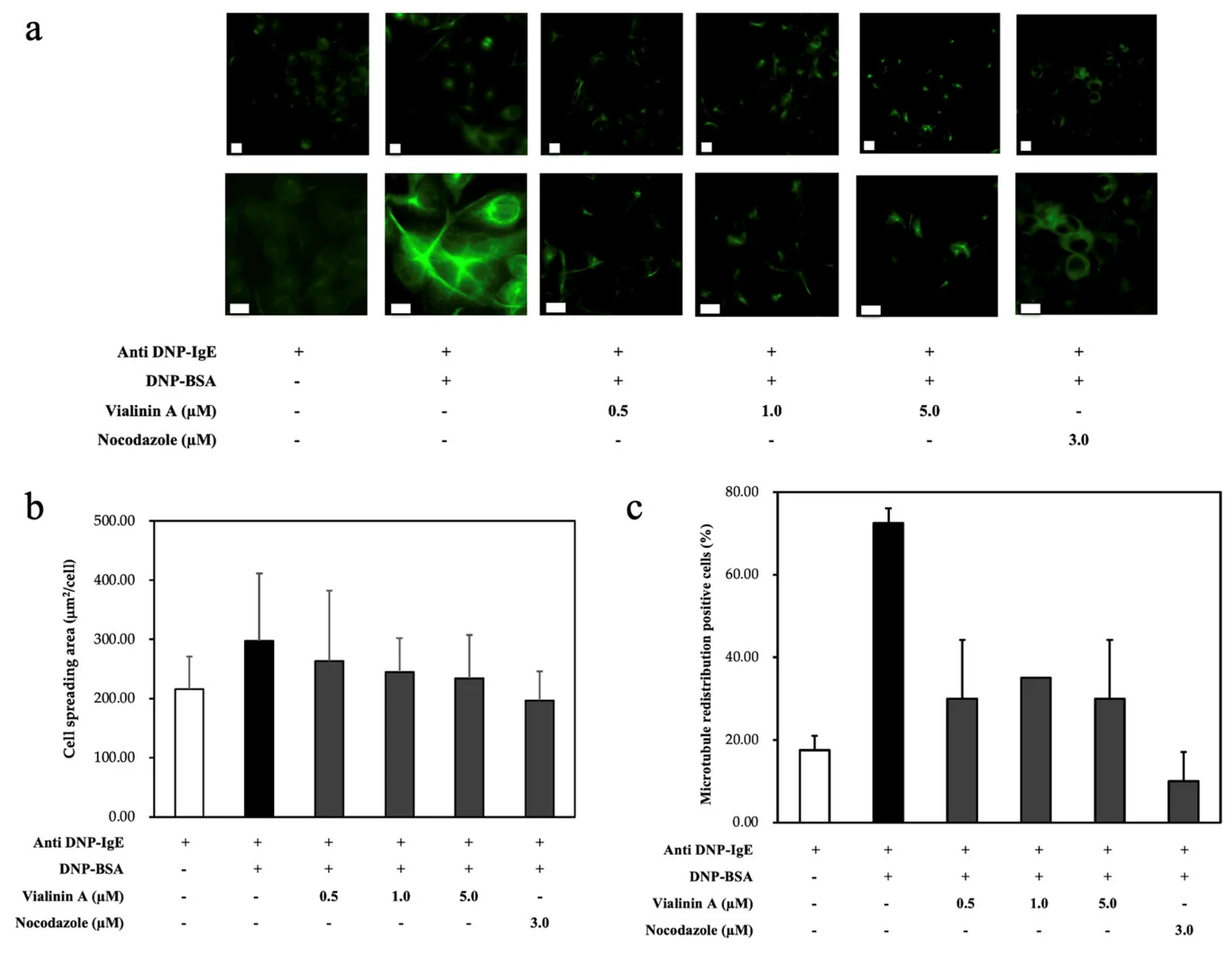
Effect of vialinin A on microtubule tract formation in DNP-IgE/DNP-BSA-stimulated RBL-2H3 cells. Degranulation in DNP-IgE-stimulated RBL-2H3 cells was initiated following treatment with various concentrations of vialinin A, DMSO as control, or 3 µM nocodazole (positive control) using DNP-BSA. Cells were fixed, permeabilized, and stained with Alexa Fluor 488-labeled anti-α-tubulin antibody (green) overnight. Cells were visualized using a Leica DMi8 optical microscope with × 20 [(**a**) upper row] or × 40 [(**a**) lower row] objective magnifications. Scale bar: 10.0 μm. (**b**) Cell spreading area was quantified using ImageJ version 1.54p. Twenty randomly selected cells per group were analyzed from two independent experiments. Data are presented as mean ± SD. Statistical comparisons were performed using one-way ANOVA followed by Dunnett’s multiple comparison test. (**c**) The percentage of cells displaying microtubule redistribution was determined from 20 randomly selected cells per treatment group.

Following the activation of PI3K/AKT, translocation to the plasma membrane accompanied by microtubule formation and secretory granules are important steps for degranulation. Thus, we investigated the effects of various concentrations of vialinin A on microtubule formation in RBL-2H3 cells following DNP-IgE sensitization and DNP-BSA stimulation. Fluorescence microscopy revealed that following DNP-BSA stimulation, microtubules spread to form tracts for secretory granules to the plasma membrane (Figure 4a). However, treatment with vialinin A effectively suppressed microtubule formation and translocation in activated RBL-2H3 cells. Quantitative analysis of cell spreading area and proportions of microtubule redistribution-positive cells demonstrated a concentration-dependent reduction in cell spreading and microtubule redistribution-positive cells following vialinin A treatment (Figure 4b,c). These analyses were performed using individual cells from two independent experiments.

In these experiments, the observed microtubule organization represents rapid cytoplasmic microtubule reorganization in interphase cells following FcεRI stimulation and should not be interpreted as mitotic spindle form.

## 3. Discussion

Allergic rhinitis has become the most prevalent type I allergy in industrialized countries, characterised by early-stage degranulation and the release of preformed mediators such as Histamine and ꞵ-hexosaminidase which stimulates mucous glands to release their secretions, inducing sneezing and nasal congestion [26,27]. A late phase response, characterized by the production and release of a variety of cytokines and chemokines like TNF-α, prolong the symptoms and may even result in complications like sinusitis and middle ear infection [28]. Impeding degranulation in mast cells has been shown to be an effective approach in attenuating allergic symptoms. However, most contemporary drugs used to treat allergic diseases, usually, anti-histamines, mast cell stabilizer or leukotriene receptor antagonists, have undesirable side effects.

Functional foods, being able to also contribute to complementary medicine, have recently been promoted over the use of drugs [29]. Vialinin A, a molecule isolated from the popular edible Chinese mushroom, *Thelephora vialis,* has been of interest to our research group. We reported its inhibitory effect on TNF-α production and release, Its inhibitory effect on Ubiquitin-Specific Peptidase 5/Isopeptidase (USP5/IsoT), and its inhibitory effect on Syk tyrosine kinase activity in RBL-2H3 cells [21,22,23,24]. In this study, its effect on other signalling molecules involved in the calcium-independent, microtubule formation degranulation pathway (Fyn–PI3K/Akt) in RBL-2H3 cells was investigated.

Fyn tyrosine kinase is a key initiator protein in this pathway. It is a non-receptor tyrosine kinase that belongs to the Src family of protein tyrosine kinases and consists of four main domains (Figure 1a). The SH4 domain, with its anchoring N-terminal, attaches to the membrane, whereas the SH3 and SH2 domains are critical in facilitating ligand binding. Finally, the bi-looped catalytic domain is responsible for kinase activity. The catalytic domain encloses a Tyr 420 phosphorylation loop and a Tyr 531 regulatory tail, whose phosphorylation regulates Fyn activity and interaction with other signaling proteins [30]. Phosphorylation of the Tyr 531 tail by C-terminal Src kinase (Csk) renders Fyn kinase inactive (closed conformation), whereas phosphorylation at the Tyr420 residue activates the kinase, allowing it to bind and phosphorylate target substrates/proteins [31]. Most Fyn inhibitors bind to the kinase’s ATP-binding site to inhibit activation and subsequent substrate phosphorylation [32]. The effects of vialinin A on Fyn activation by Tyr420 phosphorylation and subsequent substrate phosphorylation were evaluated using a Fyn kinase reaction kit (Figure 1b). Because poly(Glu:Tyr) [(Glu)_4_Tyr] is an artificial substrate, the assay evaluates the intrinsic enzymatic activity of Fyn rather than phosphorylation of a specific physiological substrate.

The results showed that vialinin A inhibited Fyn kinase activity in a dose-dependent manner. Furthermore, treatment with vialinin A at concentrations of 25.0 μM resulted in a decrease in the ratios of p-PI3K/PI3K, suggesting that the inhibition of activation associated with phosphorylation of these molecules was due to the inhibition of Fyn activation by vialinin A (Figure 1c and Figure 3a,b).

The mode of Fyn kinase inhibition by vialinin A (competitive, noncompetitive, uncompetitive, or mixed) remains to be determined. Detailed enzyme kinetic analyses using appropriate substrate and ATP concentrations will be necessary to clarify the inhibitory mechanism in future studies. To gain insight into the inhibitory mechanism of vialinin A toward Fyn kinase, molecular docking simulations were performed using AutoDock Vina. Staurosporine, used as a control compound, exhibited a predicted binding affinity of −12.4 kcal/mol, which was in close agreement with a previously reported docking score of −12.2 kcal/mol [33]. In contrast, the highest predicted binding affinity of vialinin A was −9.3 kcal/mol. Although docking scores do not directly correspond to experimentally determined binding affinities, the lower predicted affinity of vialinin A may be consistent with its weaker inhibitory potency compared with staurosporine. In our previous study, vialinin A did not exhibit inhibitory activity toward Lyn kinase. Therefore, docking simulations were also performed using Lyn kinase. Similar to the docking results obtained for Fyn kinase, vialinin A was predicted to bind within the ATP-binding site and largely overlapped with the binding position of staurosporine. Furthermore, the predicted binding affinities of vialinin A and staurosporine were comparable to those obtained for Fyn kinase. However, detailed analysis of the docking models revealed that the amino acid residues predicted to interact with vialinin A differed between Fyn and Lyn kinases. These differences in the binding mode may influence the actual binding affinity and inhibitory activity of vialinin A. Alternatively, the discrepancy between the docking results and the experimental inhibition data may reflect the limitations of docking simulations in this trial. Because the inhibitory activity of vialinin A is in the micromolar range, subtle differences in protein dynamics, solvent effects, or binding kinetics that are not fully captured by AutoDock Vina may contribute to the observed selectivity toward Fyn kinase.

In contrast, inhibition of Akt phosphorylation, as indicated by the p-Akt/Akt ratio, was observed at lower concentrations of vialinin A (5.0 μM), consistent with the greater sensitivity of PI3Kδ to vialinin A. Together with our cell-free kinase assay demonstrating direct inhibition of PI3Kδ (Figure 1d), these findings suggest that suppression of PI3Kδ signaling contributes to the inhibition of Akt activation (Figure 3a,c).

Since vialinin A also exhibited inhibitory activity toward PI3Kδ, docking analysis was performed using PI3Kδ (PDB ID: 4XE0) as the receptor. All predicted docking models of vialinin A were located in a cavity adjacent to the crystallographically observed idelalisib-binding site (Supplementary Figure S4; Site 1). Because no crystal structure of PI3Kδ in complex with ATP or an ATP analog is currently available, the binding mode was compared with that observed in the crystal structure of PI3KC2α in complex with ATP (PDB ID: 7BI6) (Supplementary Figure S4b). Based on this comparison, vialinin A was predicted to bind on the side opposite to the phosphate moiety of ATP. In the PI3KC2α structure, the corresponding cavity is unoccupied, suggesting that vialinin A may not directly interfere with ATP binding unless ligand-induced conformational changes occur.

To assess the reliability of the docking analysis, idelalisib was also subjected to docking calculations. The top-ranked docking model occupied essentially the same position as that observed in the crystal structure (Supplementary Figure S4; Site 2), whereas the second-ranked model was located in a cavity similar to that predicted for vialinin A (Supplementary Figure S4; Site 1). The third-ranked model occupied a distinct site different from both the crystallographic idelalisib-binding site and the predicted vialinin A-binding site (Supplementary Figure S4; Site 3). These results indicate that multiple energetically favorable binding poses can be generated by AutoDock Vina for PI3Kδ ligands.

Notably, the highest predicted binding affinity of idelalisib was −9.3 kcal/mol, which is comparable to those obtained for vialinin A with Fyn and Lyn kinases. However, docking scores should be interpreted with caution because they may not directly correspond to experimentally determined binding affinities or inhibitory activities.

Therefore, although the docking analyses suggested that vialinin A can bind to Fyn, Lyn, and PI3Kδ, the predicted binding affinities and poses did not fully explain the experimentally observed inhibitory profiles. In particular, similar docking scores were obtained for Fyn and Lyn kinases despite their different sensitivities to vialinin A, and the predicted binding site in PI3Kδ differed from that of the crystallographically characterized inhibitor idelalisib. These findings suggest that factors not accounted for by the docking simulations, such as protein dynamics, solvent effects, induced-fit conformational changes, or binding kinetics, may contribute to the inhibitory activity and selectivity of vialinin A. Additional structural and biochemical studies will be required to elucidate the molecular basis underlying the selectivity of vialinin A toward Fyn kinase and its inhibitory activity against PI3Kδ.

On the other hand, the reduced phosphorylation of PI3Kδ and Akt observed in cells may not be explained solely by direct inhibition of PI3Kδ. Previous studies have shown that vialinin A inhibits Syk kinase activity, indicating that inhibition of Syk-dependent signaling may also contribute to the suppression of PI3Kδ activation. In addition, the possible involvement of protein phosphatases cannot be excluded and warrants further investigation. These findings are consistent with the inhibition of microtubule formation and granule translocation following cell spreading by vialinin A observed over a similar concentration range (Figure 4a–c). Furthermore, they agree with our previous finding that vialinin A inhibits antigen-induced mast cell degranulation with an IC_50_ of 0.25–0.4 μM [24]. Overall, our findings suggest that the inhibitory effect of vialinin A on mast cell degranulation is mediated through multiple mechanisms, including direct inhibition of Fyn and PI3Kδ as well as suppression of Syk-dependent signaling, whereas the possible contribution of phosphatase-mediated regulation remains to be clarified.

Reports have shown that Gab2–PI3K/Akt are key signaling molecules in the calcium-independent microtubule formation pathway of mast cell degranulation [7,8,9,10]. Gab2 plays a key role in mediating FcεRI signals in mast cells by activating RhoA, a small protein essential in microtubule formation. Gab2 also activates and interacts with PI3K, specifically PI3Kδ, a key lipid kinase involved in mast cell maturation and degranulation [34]. Akt activation in mast cells leads to the regulated production and secretion of late-phase cytokines such as TNF-α, and the activation of their transcription factors such as the inhibitor of nuclear factor κB (IκB) and nuclear factor κB (NFκB) [35]. Immunoblotting results showed dose-dependent inhibition of phosphorylation and activation of these signaling molecules, particularly Akt, by vialinin A.

Following signal transduction of activated FcεRI, cytoskeleton reorganization is a characteristic stage in mast cell degranulation [7]. This is followed by membrane ruffling, cell spreading, movement of secretory granules to the plasma membrane, membrane fusion, and secretion of inflammatory content [11]. Changes in cell morphology following treatment with various concentrations of vialinin A were observed using fluorescence microscopy. Before antigen stimulation (blank), cells appeared to have faint microtubule tracts. DNP-BSA stimulation induces microtubule formation and spreading, promoting their translocation to the plasma membrane. Treatment with various concentrations of vialinin A suppressed microtubule formation in a dose-dependent manner, resulting in the inhibition of translocation (of secretory granules) to the plasma membrane.

**Figure 5:**
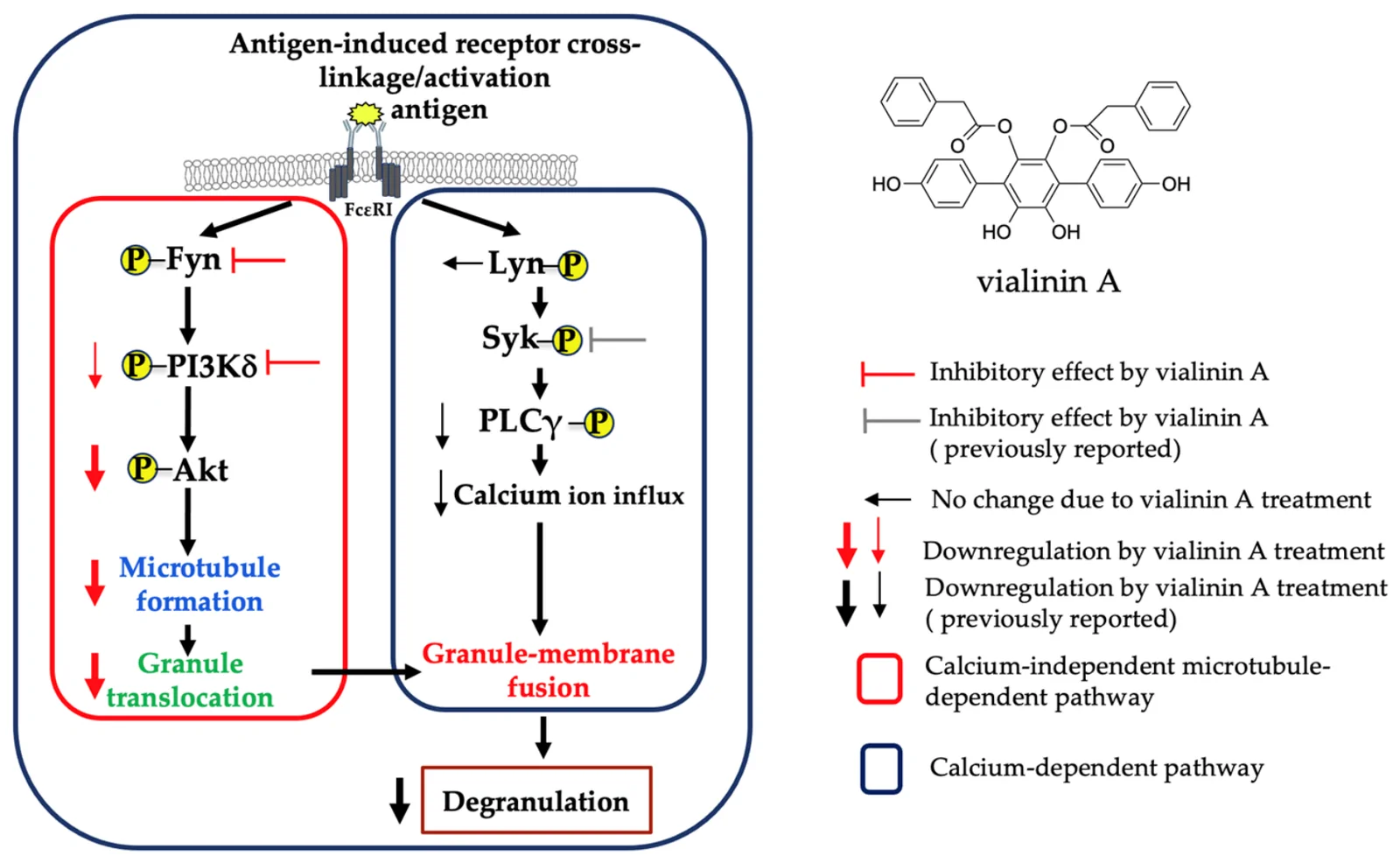
Proposed mechanism of action of vialinin A in inhibiting degranulation signaling pathways.

This study reports the inhibitory effects of vialinin A on the calcium-independent microtubule formation pathway of mast cell degranulation. As shown in Figure 5, vialinin A effectively suppresses degranulation in RBL-2H3 cells by inhibiting Fyn kinase activity and further strongly inhibiting PI3Kδ kinase activity. Vialinin A suppresses the phosphorylation of the PI3Kδ and Akt signaling molecules, thereby inhibiting microtubule formation and translocation. Our previous study demonstrated that vialinin A did not exhibit significant cytotoxicity in RBL-2H3 cells at concentrations up to 25 μM, as determined by an MTT assay [24]. Therefore, the inhibitory effects on Fyn and PI3Kδ kinase activities and Fyn/PI3Kδ signaling observed in the present study are unlikely to result from nonspecific cytotoxicity.

However, several limitations of the present study should be acknowledged. The involvement of Fyn and PI3Kδ was evaluated primarily using pharmacological inhibitors and kinase assays, and genetic approaches, such as siRNA-mediated knockdown or gene knockout, were not performed to validate the proposed mechanism. Furthermore, all experiments were conducted using a single mast cell line (RBL-2H3). Therefore, future studies using additional mast cell models, including bone marrow-derived mast cells (BMMCs) and human KU-812 cells, will be necessary to confirm the broader applicability of the present findings. In addition, a detailed investigation of the effects of vialinin A on late-phase signaling pathways, including the IκB/NF-κB axis involved in TNF-α production and release, may reveal additional mechanisms underlying its anti-allergic activity.

## 4. Materials and Methods

### 4.1. Materials

Vialinin A was purchased from Cayman Chemicals (Ann Arbor, MI, USA) and RBL-2H3 cells were obtained from the American Type Culture Collection (Manassas, VA, USA). Monoclonal rabbit antibodies against PI3K55 (11889T), p-PI3K55&85 (4228T, at Tyr199 on p55), Akt (4691T), and p-Akt (4060T, at Ser473) were purchased from Cell Signaling Technology (Danvers, MA, USA). The adenosine diphosphate (ADP)-Glo^TM^ detection system and Fyn and PI3Kδ kinase enzyme systems were purchased from Promega (Tokyo, Japan). PP2, umbralisib, nocodazole, wortmannin, and other chemicals were purchased from Fujifilm Wako Pure Chemical Corporation (Osaka, Japan).

### 4.2. Fyn Kinase Assay

The effect of vialinin A on Fyn catalytic activity was analyzed using a kinase reaction kit according to the manufacturer’s instructions, with some modifications. Fyn kinase activity was measured using poly(Glu:Tyr) [(Glu)_4_Tyr] as a synthetic substrate. Poly(Glu:Tyr) is a commonly used substrate for Src family tyrosine kinases and provides a reproducible measure of catalytic activity in vitro. Kinase activity was quantified using the ADP-Glo™ kinase assay by measuring ADP production resulting from ATP consumption during substrate phosphorylation. Specific concentrations of vialinin A and ATP were incubated with kinase for 1 h at room temperature. The ADP-Glo reagent was then added to the plate and incubated for 40 min at room temperature under light-protected conditions. Finally, the Kinase Detection Reagent was added, and the plate was incubated for 30 min at room temperature under light-protected conditions. ADP formed from the kinase reaction was measured by luminescence detection. PP2 (10 nM) was used as positive control.

### 4.3. PI3Kδ Kinase Assay

The effect of vialinin A on PI3Kδ catalytic activity was analyzed using a kinase reaction kit according to the manufacturer’s instructions, with some modifications. Specific concentrations of vialinin A and ATP were incubated with kinase for 40 min at 30 °C. The ADP-Glo reagent was then added to the plate and incubated for 45 min at room temperature under light-protected conditions. Finally, the Kinase Detection Reagent was added, and the plate was incubated for 30 min at room temperature under light-protected conditions. ADP formed from the kinase reaction was measured by luminescence detection. Umbralisib (22.2 nM) was used as a positive control.

### 4.4. Molecular Docking

The three-dimensional structures of Fyn kinase (PDB ID: 2DQ7), Lyn kinase (PDB ID: 3A4O), and PI3Kδ (PDB ID: 4XE0) were used for molecular docking calculations with AutoDock Vina. Fyn and Lyn kinase structures were determined in complex with staurosporine, whereas the PI3Kδ structure was determined in complex with idelalisib, a selective PI3Kδ inhibitor. The three-dimensional structure of vialinin A was constructed using Discovery Studio (Dassault Systèmes, Vélizy-Villacoublay, France), whereas the structures of staurosporine and idelalisib were obtained from PubChem (CID 44259 and 11625818, respectively). Prior to docking, the ligands were removed from the crystal structures of Fyn kinase, Lyn kinase, and PI3Kδ. Receptor and ligand structures were converted to PDBQT format and prepared using MGLTools Version 1.5.7 (https://ccsb.scripps.edu/mgltools/, accessed on 25 July 2026). Structural figures were generated using PyMOL Ver.2.1 (Schrödinger, LLC, New York, NY, USA).

### 4.5. Immunoblotting

RBL-2H3 cells (3 × 10^5^ cells/well) were cultured in 6-well culture plates and sensitized with 50 ng/mL DNP-IgE for 16 h. The cells were then washed with Tyrode’s-HEPES buffer (iNtRON Biotechnology, Inc., Seongnam-si, Republic of Korea), pH 7.4, and treated with 0.5 μM, 1 μM, 5 μM, 12.5 μM, and 25 μM of vialinin A or DMSO for 10 min. Wortmannin (5 μM) was used as positive control. The treated cells were subsequently stimulated with 50 ng/mL DNP-BSA for 5 min. The cell lysates were collected immediately on ice. Proteins in the total cell lysate were adjusted and separated by sodium dodecyl sulfate-polyacrylamide gel electrophoresis. Bands were transferred onto a polyvinylidene fluoride membrane and blocked with 5% skim milk dissolved in Tris-buffered saline containing 0.1% Tween 20 (TBS-T) or blocking one solution at room temperature for 1 h. Following blocking, each membrane was incubated at 4 °C overnight with a primary antibody. The next day, each membrane was washed three times with TBS-T. Membranes were incubated with horseradish peroxidase-labeled anti-rabbit IgG antibody at 24 °C for 1 h on a rocking plate. Blots were developed using ImmunoStar LD (FUJIFILM Wako Pure Chemical Corporation, Osaka, Japan) and bands were visualized using a ChemiDoc MP Plus apparatus. We visualized using image Touch software Ver. 3.0.1.14 (Bio Rad, Hercules, CA, USA). Also, the image had been adjusted to a different resolution to make it easier to see. The images were processed using ChemiDoc Imaging Systems (BIO-RAD, Hercules, CA, USA).

### 4.6. Morphological Analysis of RBL-2H3 Cells

In a 24-well flask previously button-coated with collagen for 1 h, RBL-2H3 cells were seeded and sensitized with DNP-IgE for 16 h. Cells were then treated with various concentrations of vialinin A (0.5 μM, 1 μM, or 5 μM), DMSO (negative control), or 3 µM nocodazole (positive control) for 10 min. Cells were stimulated with DNP-BSA for 10 min. The cells were fixed with 4% paraformaldehyde and permeabilized with 0.1% (*v*/*v*) Triton-X-100, in 5% BSA-PBS solution. After washing, the cells were sensitized with anti-α-tubulin conjugated with Alexa Fluor^®^ 488 for 90 min. The fluorescence levels of Alexa Fluor^®^ 488 were measured using a fluorescence microscope (Leica, DM2500, Wetzlar, Germany) [36].

### 4.7. Quantitative Analysis of Cell Spreading Area

Quantitative image analysis was performed using ImageJ (NIH, Bethesda, MD, USA). Cell boundaries were manually outlined, and the cell spreading area was measured. For each treatment group, 20 randomly selected cells were analyzed. Quantification was performed using individual cells from two independent experiments.

### 4.8. Microtubule Redistribution-Positive Cells

Cells exhibiting elongated morphology accompanied by peripheral microtubule tract formation were scored as positive for microtubule redistribution. The percentage of positive cells was calculated for each treatment group. These analyses were performed using individual cells from two independent experiments.

### 4.9. Statistical Analysis

Dunnett’s multiple comparison test was used to evaluate statistically significant differences against the control group. Statistical significance was set at *p* < 0.05. Data are expressed as mean ± SD (*n* = 3).

## Data Availability

The data presented in this study are included in the article.

## Supplementary Materials

The following supporting information can be downloaded at: https://www.mdpi.com/article/10.3390/molecules31162825/s1. References [37,38] are cited in the supplementary materials.
