# Supplementary material for: Vialinin A Inhibits Microtubule-Mediated Secretory Granule Transport for Degranulation in RBL-2H3 Cells by Targeting the PI3K/Akt Axis
Source: Molecules. 2026 Aug 13;31(16):2825. doi: 10.3390/molecules31162825 (PMC13515595; doi:10.3390/molecules31162825)
Supplement: Supplementary file 1 [file molecules-31-02825-s001.zip › molecules-4413739-supplementary.pdf]

Supplementary files

Vialinin A Inhibits Microtubule-Mediated Secretory Granule Transport for Degranulation in RBL-2H3 Cells by Targeting the PI3K/Akt Axis

Ange Murielle Djidjou Tagne [1,†], Kotoe Ishii [1,†], Yasukiyo Yoshioka [2], Kouichi Sugaya [1], Jun-ichi Onose [1], Shunsuke Yajima[3] and Naoki Abe [1,*]

[1] Department of Nutritional Science and Food Safety, Faculty of Applied Bioscience, Tokyo University of Agriculture, 1-1-1 Sakuragaoka, Setagaya-ku, Tokyo 156-8502, Japan

[2] Department of Health and Nutrition Sciences, Faculty of Human Health, Komazawa Women's University, 238 Sakahama, Inagi-shi, Tokyo 206-8511, Japan

[3] Department of Bioscience, Faculty of Life Science, Tokyo University of Agriculture, 1-1-1 Sakuragaoka, Setagaya-ku, Tokyo 156-8502, Japan

*Correspondence: abe@nodai.ac.jp (N.A.)
† These authors contributed equally to this work.

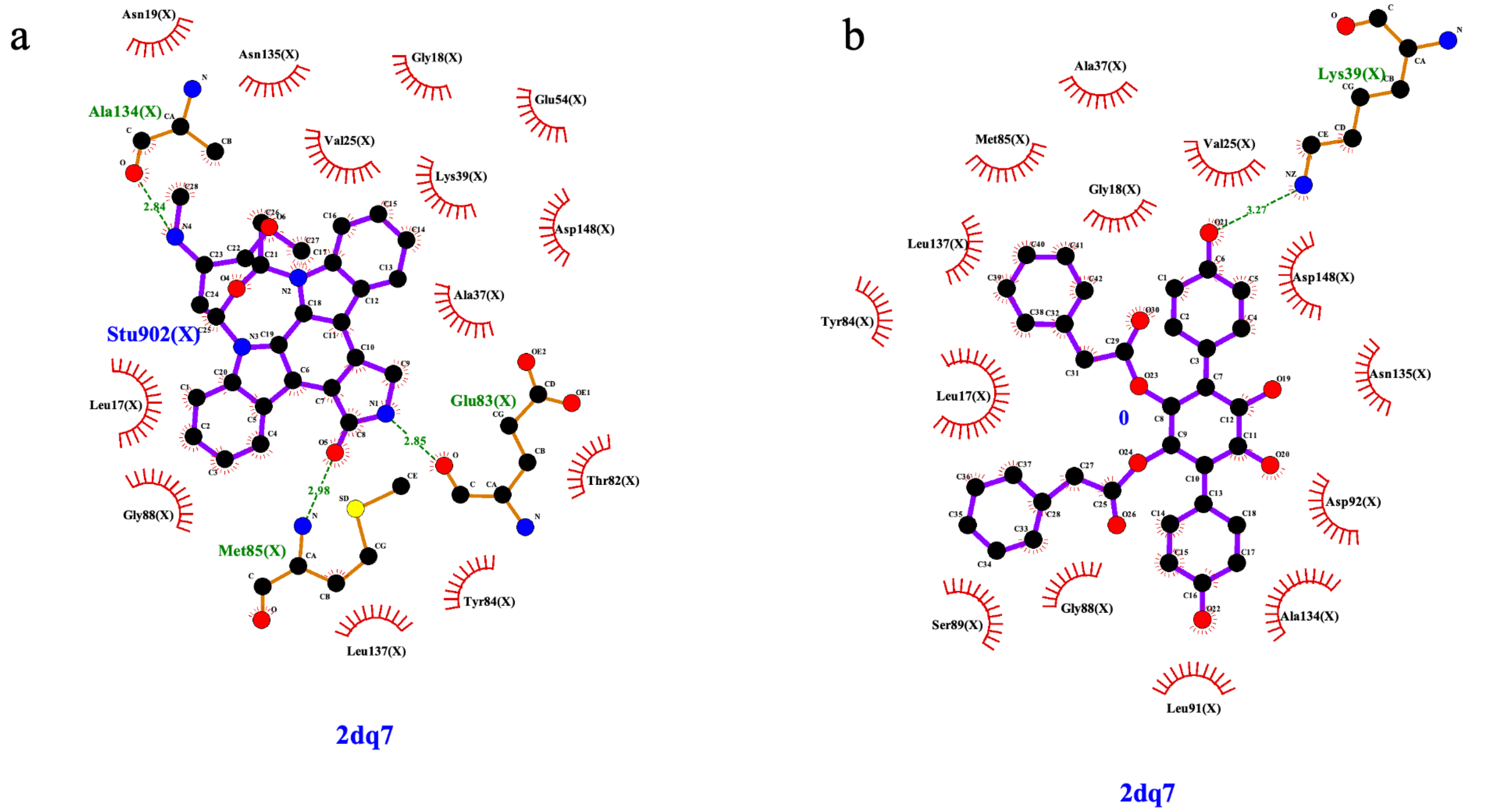

Supplementary Figure S1

Interactions between ligands and amino acid residues in the ATP-binding site of Fyn kinase.Ligand–protein interactions were analyzed using LigPlot+ [37]. The interactions of staurosporine derived from the crystal structure of the Fyn–staurosporine complex (PDB ID: 2DQ7) and those of vialinin A predicted from the docking model are shown in panels A and B, respectively. Hydrogen-bonding interactions are indicated by dashed lines, whereas hydrophobic contacts are represented by spoked arcs.

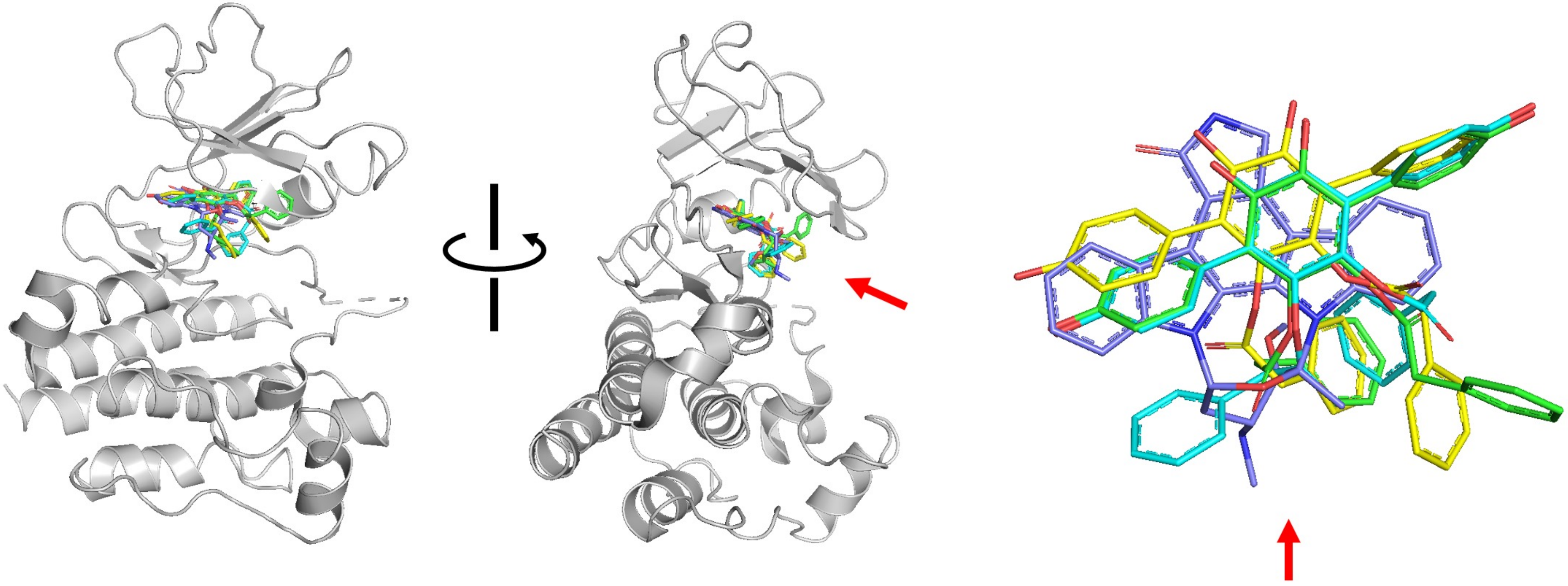

Supplementary Figure S2

Superposition of the top three docking models of vialinin A with the crystal structure of the Lyn–staurosporine complex. The top three docking models of vialinin A are shown superimposed on the crystal structure of Lyn kinase complexed with staurosporine (PDB ID: 3A4O). Front and side views are presented in the left and middle panels, respectively, with staurosporine and vialinin A displayed as stick representations. Staurosporine is colored purple, whereas vialinin A is colored cyan, green, and yellow in order of decreasing predicted binding affinity. The right panel shows only the bound molecules. Red arrows in the middle and right panels indicate the viewing direction of the compounds.

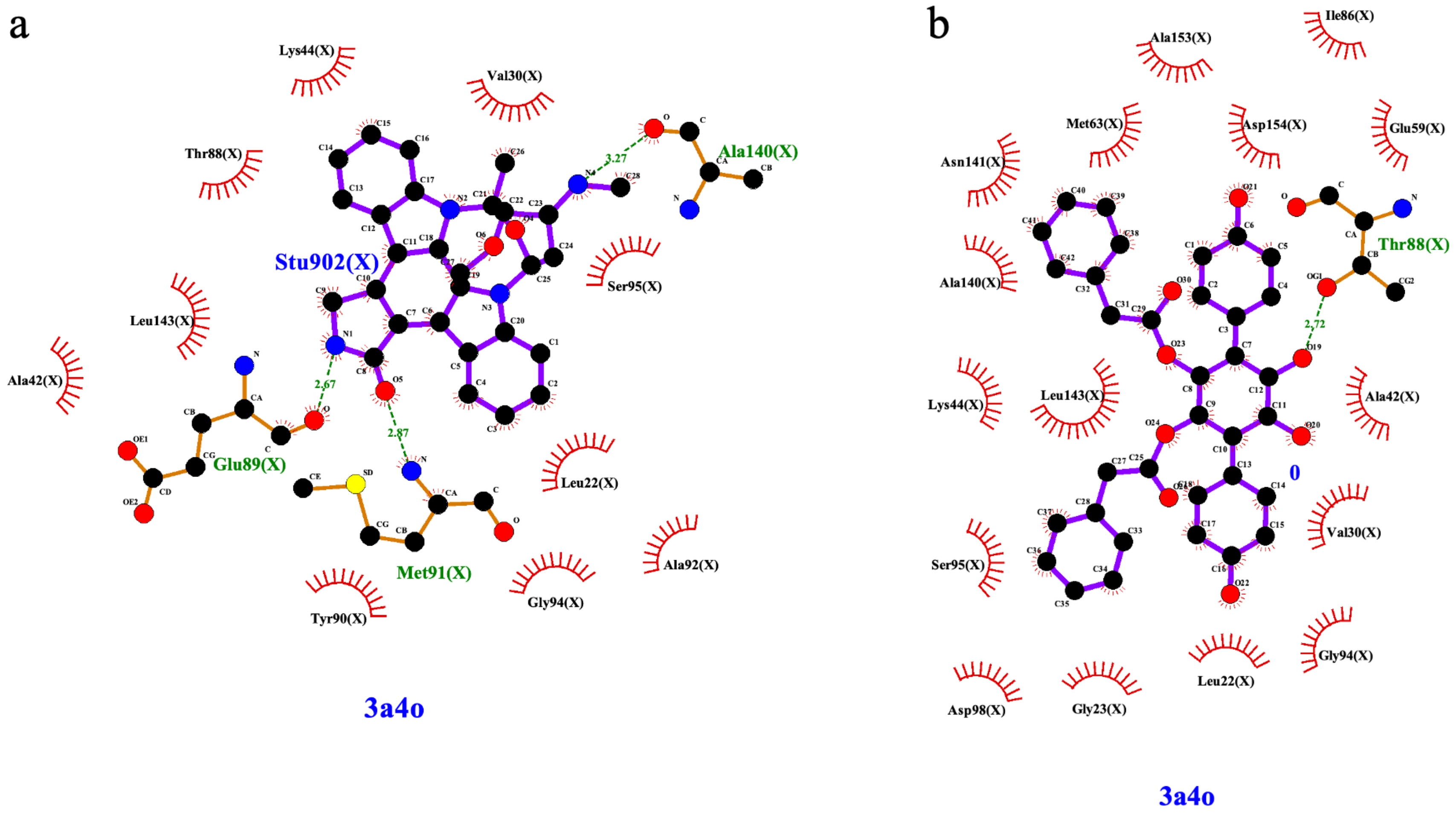

Supplementary Figure S3

Interactions between ligands and amino acid residues in the ATP-binding site of Lyn kinase. The analysis was performed as described for Supplementary Fig. 1 using Lyn kinase (PDB ID: 3A4O) instead of Fyn kinase. The staurosporine and predicted interactions of vialinin A with Lyn kinase are shown in panels A and B, respectively. Hydrogen-bonding interactions are indicated by dashed lines, whereas hydrophobic contacts are represented by spoked arcs.

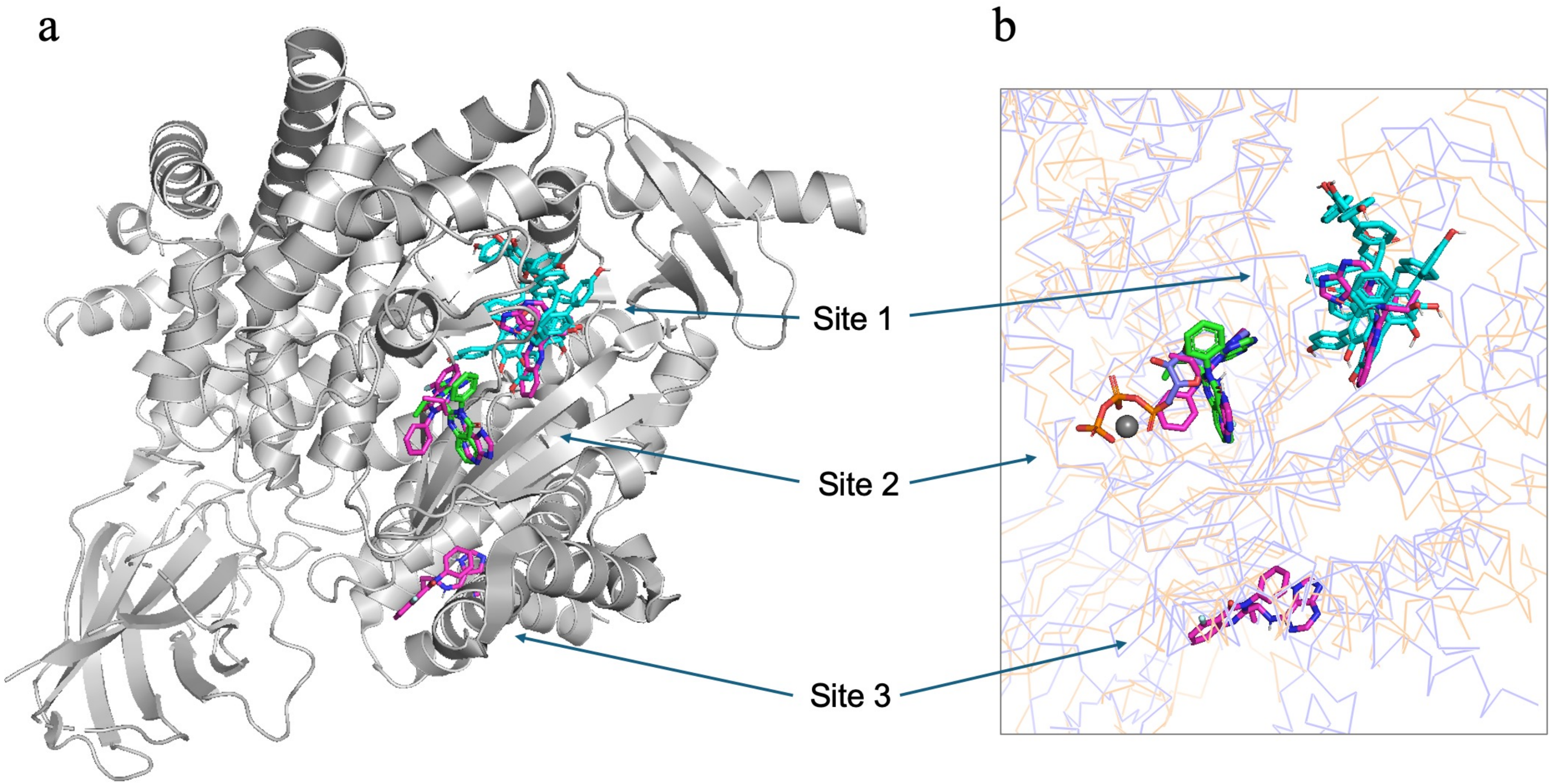

Supplementary Figure S4

Predicted binding sites of vialinin A and idelalisib in PI3Kδ. (a) The top three docking models of vialinin A and idelalisib were superimposed on the crystal structure of PI3Kδ in complex with idelalisib (PDB ID: 4XE0). Predicted vialinin A, predicted idelalisib, and crystallographically observed idelalisib are shown in cyan, red, and green, respectively. (b) PI3KC2α (PDB ID: 7BI6) was superimposed on PI3Kδ (PDB ID: 4XE0) using the MatchMaker function implemented in UCSF Chimera [38]. The backbone structures of PI3KC2α and PI3Kδ are shown as ribbon representations colored light orange and light purple, respectively. Predicted vialinin A, predicted idelalisib, crystallographically observed idelalisib, and ATP from the PI3KC2α–ATP complex are colored cyan, red, purple, and green, respectively. The grey colored sphere represents Mg^{2+} bound to ATP. The binding sites of predicted vialinin A, crystallographically observed idelalisib, and the third-ranked docking model of idelalisib are designated Sites 1, 2, and 3, respectively, as described in the main text.
